# Epidural Blood Patch in a Patient with a Hematological Malignancy

**DOI:** 10.1155/2023/9955772

**Published:** 2023-02-10

**Authors:** Ross Barman, Jack McHugh, Thomas O'Mara, Thomas Pittelkow, Ryan S. D'Souza

**Affiliations:** ^1^Department of Anesthesiology & Perioperative Medicine, Mayo Clinic, Rochester, MN, USA; ^2^Department of Internal Medicine, Mayo Clinic, Rochester, MN, USA; ^3^University of New England College of Osteopathic Medicine, Biddeford, ME, USA; ^4^Department of Anesthesiology & Perioperative Medicine, Division of Pain Medicine, Mayo Clinic, Rochester, MN, USA

## Abstract

Postdural puncture headache is a frequently encountered complication following procedures such as lumbar puncture, neuraxial anesthesia, or intrathecal drug delivery device implantation. It classically presents as a painful orthostatic headache that is exacerbated when a patient is upright. For treatment, patients are often started on conservative options such as hydration, caffeine, bedrest, and NSAID analgesics; however, certain patients who fail these therapies may require intervention with an epidural blood patch. The epidural blood patch remains the gold standard for treating refractory postdural puncture headache. Contraindications to epidural blood patch include severe coagulopathy, patient refusal, or infection at the intended site of entry. There are no clear consensus recommendations regarding patients with a hematological malignancy and potential risk that autologous blood may seed malignant cells into the neuraxis. In this case report, we present a patient with acute myeloid leukemia who developed a postdural puncture headache after receiving subarachnoid administration of antineoplastics. The patient was refractory to conservative therapy, prompting multidisciplinary consultation and discussion with the patient about the risks and benefits of proceeding with an epidural blood patch. Ultimately, the patient elected to proceed with the offered epidural blood patch which led to complete resolution of his painful headaches and did not cause any spread of malignant cells into his neuraxis or cerebral spinal fluid.

## 1. Introduction

Postdural puncture headache (PDPH) is a frequently encountered complication following any neuraxial procedure which either purposefully or inadvertently punctures the dura. Risk factors for PDPH include age less than 60, female gender, pregnancy, and the use of larger gauge or cutting needles [1]. A dural puncture headache presents as a severe fronto-occipital headache which is orthostatic in nature, namely, symptoms are exacerbated when the patient is upright and improved when laying supine. The pathophysiology is debated; one proposal posits that upright positioning causes increased loss of cerebral spinal fluid (CSF) and intracranial sagging which elicits painful traction on the meninges, while an alternative mechanism suggests pain is elicited from meningeal vasodilation which is a compensatory mechanism for intracranial hypotension [2, 3]. Conservative treatment includes hydration, caffeine, bedrest, and abdominal binder application, along with medications such as non-steroidal anti-inflammatory drugs (NSAIDs) or acetaminophen. For patients refractory to conservative treatments, an epidural blood patch (EBP) remains the most effective therapy with greater than 75% of patients reporting complete symptom resolution [1]. EBP contraindications include coagulopathy, elevated intracranial pressure, patient refusal, bacteremia, or local infection at the puncture site. In patients who are unable to receive an EBP, a sphenopalatine ganglion block via topical anesthetic may also provide relief.

An EBP is usually considered a low-risk procedure when autologous blood from a healthy patient is used; however, further consideration is warranted in a patient with a hematological malignancy or with immunocompromised status. The literature on this subject is sparse, and no consensus or expert recommendations exist to guide clinicians. While alternatives to autologous blood, such as donated allogenic blood, fibrin glue, or saline have been postulated, there is a paucity of data on their efficacy.

## 2. Case Report

A previously healthy 31-year-old male with a medical history only significant for previous polysubstance abuse presented to his local emergency department with a one-month history of night sweats, fatigue, and dyspnea on exertion. Emergency department workup revealed a critical leukocytosis of 466 × 10^9^/L (normal range = 4.5–11 × 10^9^/L), normocytic anemia with hemoglobin of 4.1 g/dL, and thrombocytopenia with a platelet count of 18 × 10^9^/L (normal range = 150–400 × 10^9^/L). Given concern for acute leukemia with impending blast crisis, the patient was emergently transferred to a tertiary medical center for treatment escalation. Cytoreductive therapy was initiated, and subsequent bone marrow biopsy revealed a diagnosis of acute myeloid leukemia (AML). Induction chemotherapy was initiated, and on hospital day 28, a diagnostic lumbar puncture (LP) was performed to examine for central nervous system disease. A 20-gauge cutting spinal needle was inserted at the L3-L4 level to obtain a CSF sample, and no immediate postoperative complications were noted. Wright-Giemsa staining of CSF showed blast cell formation, and intrathecal (IT) chemotherapy was initiated on day 31, and the intrathecal space was again accessed via a 20-gauge cutting spinal needle. Less than 24 hours after this procedure, our patient reported a debilitating positional headache that was brought on after sitting upright or standing for 30 minutes. He rated the pain intensity as a 9 out of 10 when upright, with complete resolution when recumbent. He endorsed that the headaches were accompanied by mild nausea but denied vision changes, photophobia, fever, and neck stiffness, and he had no focal neurologic deficits on exam. He was initiated on conservative therapy with both oral and parenteral caffeine, intravenous fluids, and acetaminophen. After 11 days of intractable postural headache pain, the Interventional Pain Service was consulted for consideration of an EBP.

**Figure 1:**
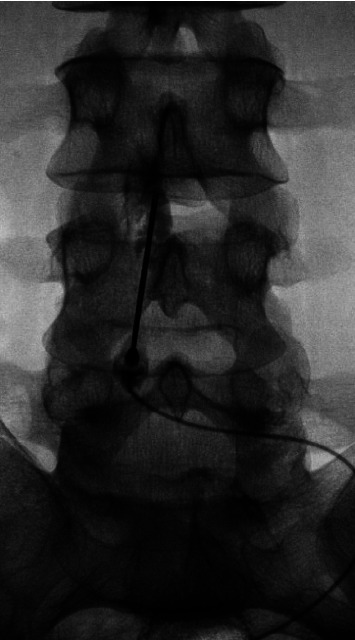
The L3-L4 epidural space was accessed under fluoroscopy, and correct placement was confirmed with injection of contrast.

Prior to consenting the patient for EBP, the team considered the possibility of meningitis, in addition to the risk of seeding malignant cells into the CSF. Given the presence of the characteristic postural headache and the absence of fever over the previous 11 days, the probability of meningitis was deemed to be very low. With respect to the consideration of the risk of seeding the CSF, it was noted that in contrast to the most recent CSF analysis, repeat bone marrow biopsy one day prior did not identify blast cells. After a thorough, multidisciplinary discussion ensuring his understanding of the risks, the patient decided to pursue EBP. Under fluoroscopic guidance, an 18G Tuohy needle was used to access the L3-4 epidural space via loss-of-resistance technique. Fluoroscopic contrast injection confirmed epidural placement, and the patient received a slow injection of 20 mL autologous blood (Figure 1). The procedure was uncomplicated, and within an hour the patient had complete resolution of symptoms. Following the successful EBP, he had three additional, uncomplicated lumbar punctures for both CSF blast cell analysis and ongoing IT chemotherapy. The subsequent CSF findings were negative for blast cells, and he did not have any further postural headaches. At time of follow-up one year later, the patient had achieved remission, remained free from substance use, and had no recurrence of postural headaches.

## 3. Discussion

The International Classification of Headache Disorders' criterion for a PDPH diagnosis is the development of a headache within 5 days of dural puncture that either spontaneously resolves within 1 week or within 48 hours after EBP [2]. A postural headache which worsens when upright is pathognomonic for PDPH, and the most common associated symptoms include nausea, neck stiffness, and rarely photophobia [2]. When evaluating a patient with a postural headache after a dural puncture, a thorough history and physical exam should be performed to rule out more insidious etiologies masquerading as PDPH, such as posterior reversible encephalopathy, subdural hematoma, cerebral venous thrombosis, or arachnoiditis [4, 5].

Conservative management should be considered and discussed with the patient, as approximately 50% of dural puncture headaches will resolve within 4 days of dural puncture [2]. EBP remains the gold standard of treatment for PDPH, with resolution rates >75% [6]. There are limited data on the risk of seeding malignant cells into the neuraxis with autologous blood from a patient with an active hematological malignancy; thus, the decision to perform an EBP in these patients remains challenging.

Albeit sparse, there is literature addressing whether a patient with an active hematological malignancy is a candidate for EBP given the potential risk to seed the neuraxis with autologous blood containing malignant cells. Strand et al. retrospectively reported outcomes of 19 hematologic malignancy patients who underwent EBP and found that one patient with diffuse large B-cell lymphoma (DLBCL) subsequently developed a biopsy-confirmed DLBCL brain lesion following EBP [7]. However, the authors suggest that its location on the corpus callosum make the epidural or intrathecal spaces an unlikely anatomical point of origin [7]. Demaree et al. conducted a retrospective review of 18 leukemia and 62 lymphoma patients who underwent EBP for PDPH, and after a median follow-up period of over 3 years, they reported that no patients developed CNS involvement [8].

Providers should be cognizant of the infection risk associated with EBP administration, particularly in the immunocompromised host. Seeding of normal skin flora into the CSF or epidural space during catheter insertion may lead to meningitis or epidural abscess formation, respectively [9]. Contamination of the injection site by the normal upper airway flora of the proceduralist has also been implicated in infection following neuraxial interventions. Each of these modes of transmission can be minimized by meticulous attention to aseptic technique. Hand hygiene, use of sterile gloves and a facemask, and decontamination of the injection site with an alcohol-based antiseptic should be routine aspects of aseptic technique in neuraxial anesthesia [10]. A further mode of transmission that deserves special consideration in this case is the direct inoculation of microorganisms contained in the blood patch where the patient has an underlying blood stream infection (BSI). On the basis of expert consensus, BSI is a relative contraindication to neuraxial anesthesia [10]. A neutropenic host may not manifest with the usual signs of BSI, and in cases where a patient is febrile and has an absolute neutrophil count <500 cells/*μ*L, the procedure should be postponed.

In this case report, we described a patient with a PDPH who was hospitalized for a recently discovered hematological malignancy. The patient continued to have debilitating postural headache pain eleven days after his most recent lumbar puncture, and he had failed conservative treatments. In our case, the decision to proceed with an EBP was influenced by a serendipitous finding that on the day prior, his bone marrow biopsy was bereft of blast cells while they were still detected in his CSF analysis. While these findings do not eliminate the risk of CNS seeding, they helped tilt the risk-benefit analysis in favor of proceeding with an EBP.

Although definitive solutions are not always present in such challenging medical situations, it is imperative for the clinician to have an open discussion with the patient on risks and benefits of EBP and to assess the degree of discomfort and burden from the PDPH. Given the lack of safety data and the considerable variability in the goals each patient may have, we advocate for a multidisciplinary discussion individually tailored to the needs of the patient.

## Data Availability

No data were used to support this study.
